# Functional analysis of a putative HER2-associated expressed enhancer, Her2-Enhancer1, in breast cancer cells

**DOI:** 10.1038/s41598-023-46460-x

**Published:** 2023-11-09

**Authors:** Mahdieh Rojhannezhad, Bahram M. Soltani, Mohammad Vasei, Nassim Ghorbanmehr, Seyed Javad Mowla

**Affiliations:** 1 Department of Molecular Genetics, Faculty of Biological Sciences, Tarbiat Modares University, Tehran, Iran; 2 Cell-Based Therapies Research Center, Digestive Disease Research Institute, Shariati Hospital, Tehran University of Medical Sciences, Tehran, Iran; 3 Biotechnology Department, Faculty of Biological Sciences, Alzahra University, Tehran, Iran

**Keywords:** Cancer, Cell biology, Genetics, Molecular biology

## Abstract

HER-2/neu (HER2) is a member of the epidermal growth factor receptors family, encoding a protein with tyrosine kinase activity. Following the gene amplification or increased HER2 transcription, carcinogenesis has been observed in some cancers. Genetic and epigenetic changes occurring in enhancer sequences can deeply affect the expression and transcriptional regulation of downstream genes, which can cause some physiological and pathological changes, including tumor progression. A therapeutic approach that directly targets the genomic sequence alterations is of high importance, with low side effects on healthy cells. Here, we employed the CRISPR/Cas9 method to genetically knockout an expressed putative enhancer (GH17J039694; we coined it as Her2-Enhancer1) located within the HER2 gene, 17q12: 39,694,339–39,697,219 (UCSC-hg38). We then investigated the potential regulatory effect of Her2-Enhancer1 on HER2 and HER2-interacting genes. To evaluate the *cis* and *trans* effects of Her2-Enhancer1, genetic manipulation of this region was performed in HER2-positive and -negative breast cancer cells. Our bioinformatics and real-time PCR data revealed that this putative enhancer region is indeed expressed, and acts as an expressed enhancer. Further functional analysis on edited and unedited cells revealed a significant alteration in the expression of HER2 variants, as well as some other target genes of HER2. Moreover, the apoptosis rate was considerably elevated within the edited cells. As we expected, Western blot analysis confirmed a reduction in protein levels of HER2, GRB7, the gene interacting with HER2, and P-AKT in the PI3K/AKT pathway. Altogether, our findings revealed an enhancer regulatory role for Her2-Enhancer1 on HER2 and HER2-interacting genes; and that this region has a potential for targeted therapy of HER2-positive cancers.

## Introduction

ErbB2, or human epidermal growth factor receptor (HER2/neu), is a member of the EGFR tyrosine kinase receptor family responsible for vital signaling pathways in normal and malignant breast epithelial cells^1^. The protein structure of HER2 comprises a ligand-binding extracellular, an intermembrane, and an intracellular tyrosine kinase domain. Extracellular signaling is triggered by changing the spatial structure through other heterodimeric proteins bound to the ligand^2,3^. *HER2* amplification is an important mechanism for its overexpression, however, high rates of *HER2* transcription per each copy of the gene have also been observed in breast cancer cells with increased *HER2* amplification^4^. *HER2* amplification has both predictive and prognostic value for breast cancer development^5^. Changes in copy numbers of genes such as *ERBB2* have been extensively documented in breast cancer cells and numerous model cell lines^6^. HER2-positive breast cancer accounts for about 25% of all cases and implies a poor prognosis. Although progress has been made in understanding HER2 signal transduction, little is known about how HER2 is subjected to gene regulation^7^. Increased *HER2* expression in HER2-positive breast cancer (HER2+) cells causes loss of polarity and cell adhesion, leading to the onset of phosphorylation events and activation of TGFb/SMAD pathway through RAS signal cascade, increased signaling activities, and expression of HER2 variants^2,3^. The level of gene transcription in HER2+ breast cancer cells is proportionally correlated with the amplification level of the gene^4^. The association of HER2 overexpression with several human tumors, extracellular access and its role in cell cycle, apoptosis, EMT, and tumor invasion^2,3,8^ are all important factors making this receptor an appropriate target for cancer therapy. Sequence and copy number changes in regulatory elements, enhancers, and promoters, are common causes of gene dysregulation in cancers^8^. Enhancers are short DNA motifs acting as binding sites for sequence-specific transcription factors which can be hundreds of kbs away from their target gene^9,10^. The eRNAs are transcribed non-coding RNAs of functional enhancers associated with super-enhancer regions. Super-enhancers, a class of enhancers regulating cell-specific genes, contain significant regulatory DNA sequences covered by transcription factors, histone markers, and co-activators. The eRNAs indicate the activation of surrounding genes rich in H3K4me1 and are also involved in the transcription of nearby genes. Transcription of eRNAs from enhancers occurs in different cell types in response to different stimuli. In addition to the gene-regulating role linked to enhancers, enhancer-resulting transcribed eRNAs are also involved in a variety of biological functions. Numerous erythrocyte-specific eRNAs, for instance, play a role in the maturation of red blood cells^11,12^.

*HER2* is located at 17q12 (Ref-Seq: NG_007503) with several transcripts including two groups of long and short variants. Two promoters and two enhancers have been reported so far, as the regulatory elements of *HER2* expression^13^. Due to the importance of HER2 and its overexpression and amplification in breast and some other cancers, diagnosis, and treatment of HER2+ tumors are of vital clinical importance. Here, we have investigated the expression, *cis*, and *trans*-regulatory effects, and function of a predicted HER2-associated enhancer, Her2-Enhancer1. Our data revealed a novel expressed enhancer within the *HER2* with regulatory effects on HER2 variants and HER2-related genes, as well as an anti-apoptotic function on HER2-positive cells.

## Results

### Predicting a putative enhancer located within the HER2 gene

**Figure 1:**
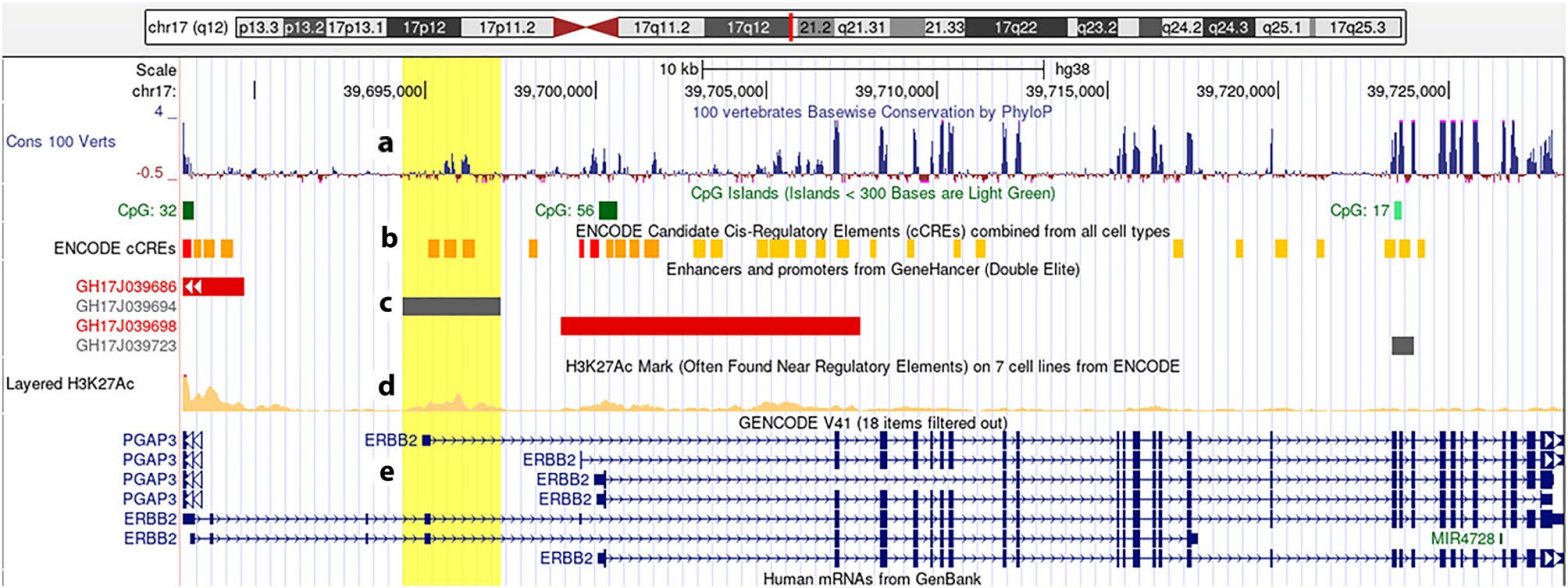
Location of Enhacer1-Her2, HER2 variants, promoters, and enhancers. (**a**) The extent of HER2 conservation among vertebrates. (**b**) *Cis* Regulatory Elements. (**c**) The location of Enhacer1-Her2 in the UCSC browser is highlighted in the figure. (**d**) Histone status of Enhacer1-Her2 region. Histone marks such as H3K27Ac (that is shown here) and H3K4me1 are often present next to regulatory elements. (**e**) Transcript variants of ERBB2.

HER2 gene contains 27 coding exons that translate into a 185 kDa transmembrane glycoprotein with 1255 amino acids. A putative HER2 segment, GH17J039694, with a size of 2881 bp located at position chr17: 39,694,339–39,697,219 (UCSC-hg38) of the gene, is suggested as an enhancer regulatory region in databases including ENCODE (Z-Lab), Ensembl, dbSUPER, GeneCards and Craniofacial Atlas (GRCh38/hg38). This predicted enhancer region, so-called Her2-Enhancer1 (Her2-En1), which starts from intron 3 and continues to the middle of intron 4 (based on NM_001289936.2), contains some features of the regulatory elements (Fig. 1). There are three patches of conserved sequences within the putative Her2-En1 region. These conserved sequences are overlapped with three DNase hypersensitive sites of the ENCODE registry of candidate *cis*-regulatory Elements (cCREs, Fig. 1b). The region exists as an open chromatin in the embryonic stem cell lines such as H1-hESC, H7-hESC as well and HepG2 hepatocellular carcinoma cell line (UCSC GRCh38/hg38). These features suggest an enhancer role for this DNA segment of the gene (Fig. 1c). According to ENCODE data, histone modifications such as H3K4me3 and H3K27ac indicate the promoter regions and histone marks such as H3K4me1 and H3K27ac are indicative of enhancer regions. The Her2-En1 region contains the histone marks; H3K4me1 and H3K27ac, which indicate an open chromatin status in the enhancer region (Fig. 1d). The putative enhancer is located upstream of a set of short variants of HER2 or group 2 variants ($\cong$ 2-kb upstream of promoter 2), and hence could regulate the expression ratio of the short/long transcripts of the gene (Fig. 1e). According to TF ChIP- seq data in UCSC, several transcription factors such as HDAC, POLR2A/G, and EP300 bind to this region in different cell lines such as H1-hESC and HEPG2. The possible role of this region in GRB7, PGAP3, NEUROD2, ORMDL3, MIEN1, and LRRC3C genes has also been reported as a super-enhancer in some cell lines (ENCODE/UCSC: Based on 3C data (Fig. S1)).

### The Her2-En1 is expressed and probably acts as an expressed enhancer

**Figure 2:**
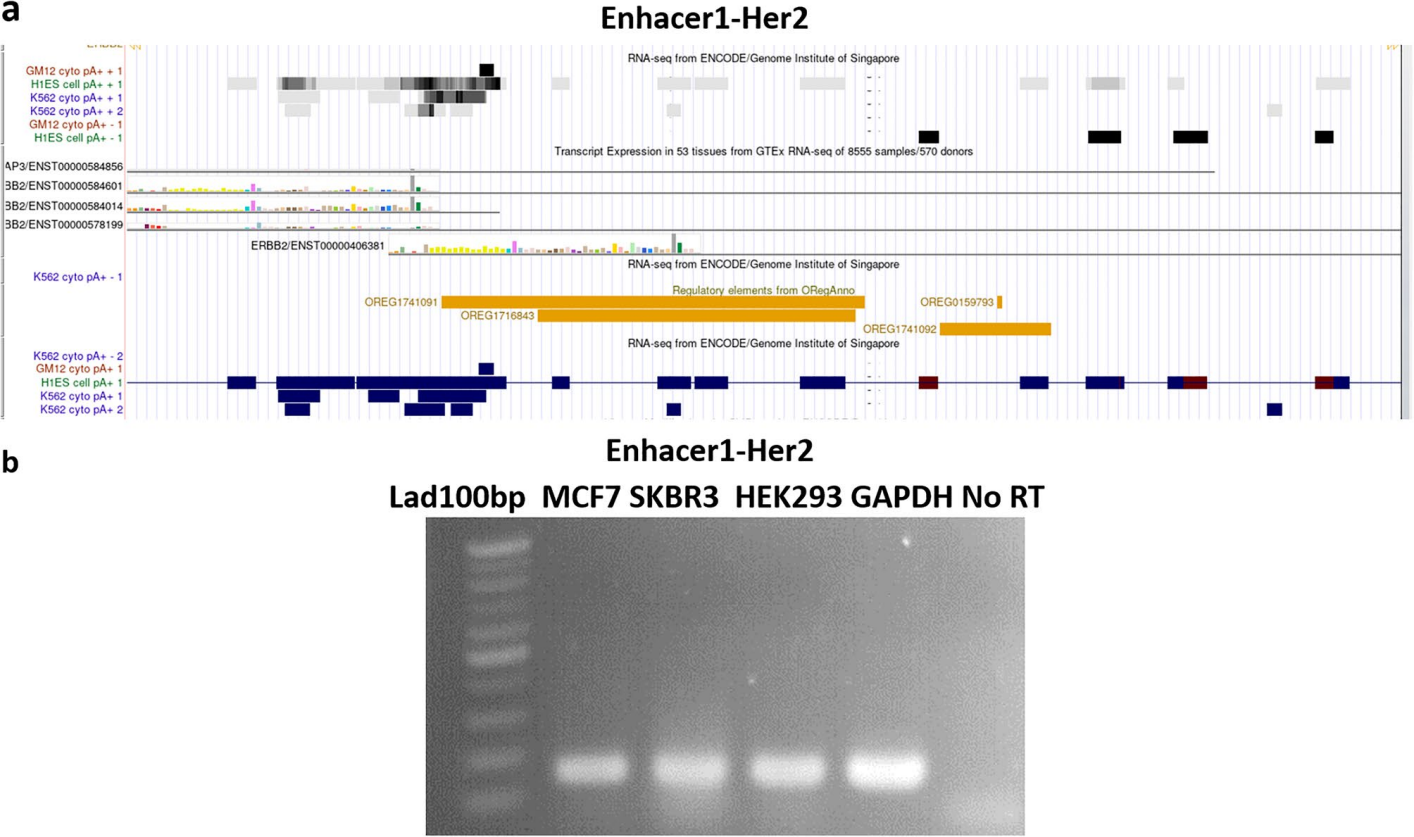
RNA-seq from ENCODE/Genome Institute of Singapore and Transcript Expression in 53 tissues from GTEx (UCSC). (**a**) The expression status of the studied region as eRNA or enhancer short reads at various positions provides valuable insights into the regulatory activity. (**b**) HER2-eRNA expression (HER2-expressed enhancer) in three cell lines with primers for a region of enhancer DNA. The primers are listed in Table 1.

Since some enhancers are transcribed to produce enhancer RNAs (eRNAs), we employed the RNA-seq from ENCODE/Genome Institute of Singapore, and Transcript Expression in 53 tissues from GTEx of UCSC and found some reads for the enhancer region (Fig. 2a). Then, specific primers were used for Her2-En1 region amplification using RT-PCR on total RNA extracted from HEK293, SKBR3, and MCF7 cells that were pre-treated with DNase enzyme. Results indicated that expected transcripts are produced from the Enhacer1-Her2 region in all tested cell lines (Fig. 2b) but there was no expression in No RT or negative control. Overall, both bioinformatics and experimental data verified the production of eRNA from the putative enhancer sequence.

### Functional suppression of Her2-En1 by CRISPR/Cas9 system

**Figure 3:**
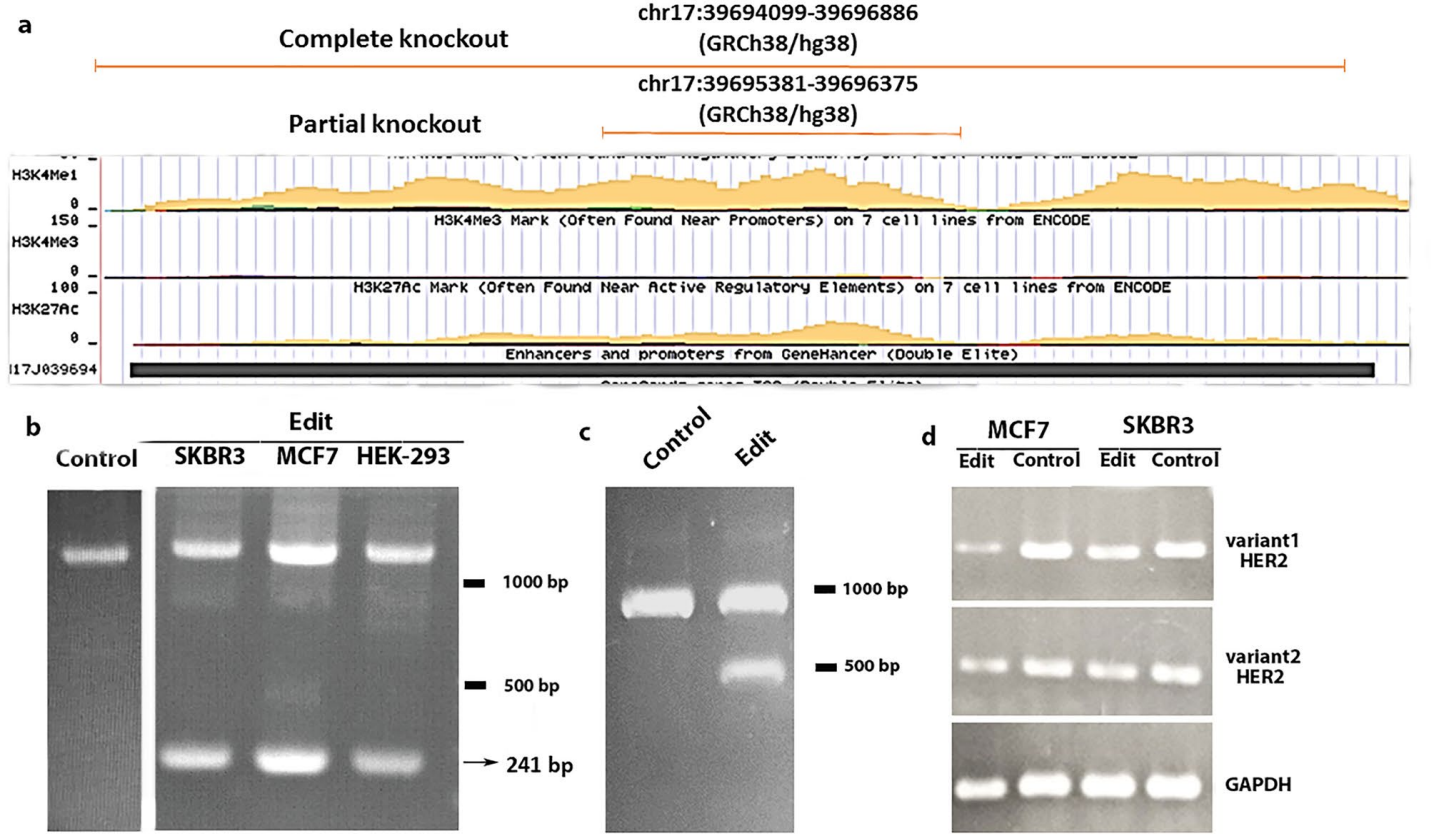
PCR Edit-test. (**a**) Schematic view of edited areas of HER2-eRNA with partial and complete knockout of Her2-En1 at UCSC. (**b**) Both unedited (1239 bp) and edited (241 bp) bands are shown (partial knockout). Note: A photo of two parts of the gels is given side by side. (**c**) Complete knockout of Her2-En1 with edit band at 482 bp. An un-edit band of approximately 1000 bp can also be seen in the figure. (**d**) RT-PCR with GAPDH as an internal control and HER2 variants 1 (214 bp) and 2 (156 bp) in SKBR3, MCF7. Wells 1 and 3 from the left, indicated reduced expression of variants due to the editing of the predicted enhancer.

We used two sets of gRNAs for complete (2787 bp) or partial (998 bp) removal of genomic sequences corresponding to Her2-En1 (Fig. 3a). Following the transfection of the cells with recombinant vectors expressing specific gRNAs, PCR was performed with a pair of edit-test primers located on either side of the editing area to determine the occurrence of genome editing (Fig. 3b,c). The PCR results were subsequently confirmed by sequencing showing the accurate deletion of the region (Fig. S2). Also, DNA copy number assessment using Real-Time PCR and standard curve plot indicated reduced copy numbers of the candidate sequences, after editing.

We also determined a possible *cis*-regulatory role of Her2-En1 on the expression levels of HER2 short and long variants (here, group 1 and 2 variants), using RT-PCR (Fig. 3d). As is shown for the MCF7 cell line, the expression levels of both short and long variants are noticeably declined in edited cells, compared to unedited cells. There was a similar but subtle down-regulation effect in the SKBR3 cell line (Fig. 3d), probably due to a higher copy number of *HER2* in these cells (Fig. S3 and Table S1).

### The *cis* and *trans*-regulatory effects of Her2-En1

**Figure 4:**
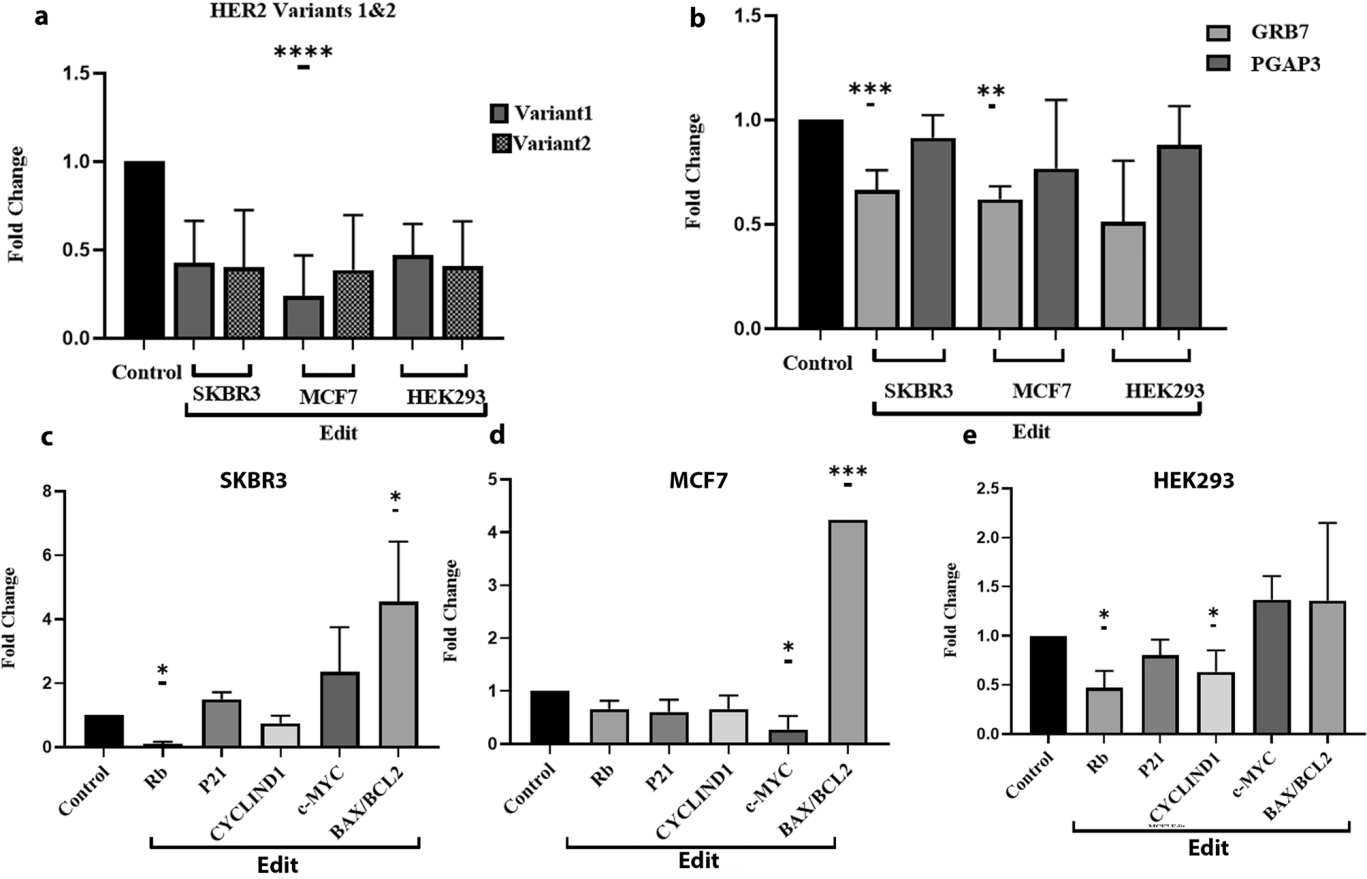
The *cis* and *trans* roles of Her2-En1. (**a**) The analysis of two groups of variants in different cell lines. In both groups of variants, a significant decrease (*P* value: < 0.0001) was observed in three cell lines. (**b**) The analysis of genes interacting with HER2, PGAP3, and GRB7 in different cell lines. Decreased expression in *GRB7* indicates a possible association between this gene’s expression and HER2 expression (although, no significant decrease was observed in the HEK293 cell line). (**c**–**e**) The analysis of genes involved in cell cycle, proliferation, and apoptosis in three cell lines. (Significance level was considered less than 0.05). *: 0.01, ****: < 0.0001.

The expression profile of HER2 variants in RNAs extracted from different cell lines was evaluated quantitatively and semi-quantitatively with specific primers for variants 1 and 2. As shown in Fig. 4a, both groups of variants showed reduced *HER2* expression compared to the control group in all three cell lines. Figure 4b shows an overriding decrease in *GRB7* in SKBR3 and MCF7 cell lines. A reduction in *Rb* expression was also observed in both SKBR3 and HEK293 cells, and *c-Myc* showed a considerable downregulation only in MCF7 cells. A substantial downregulation of *CCND1* (cyclin D1), a gene effective in cell cycle progression, was also noticed in the HEK293 cell line. Moreover, the upregulation of the *BAX/BCL2* ratio in SKBR3 and MCF7 cell lines suggested a potential elevation in the rate of apoptosis in edited cells (Fig. 4c–e). GAPDH was used as an internal control and the significance level was considered as less than 0.05.

**Figure 5:**
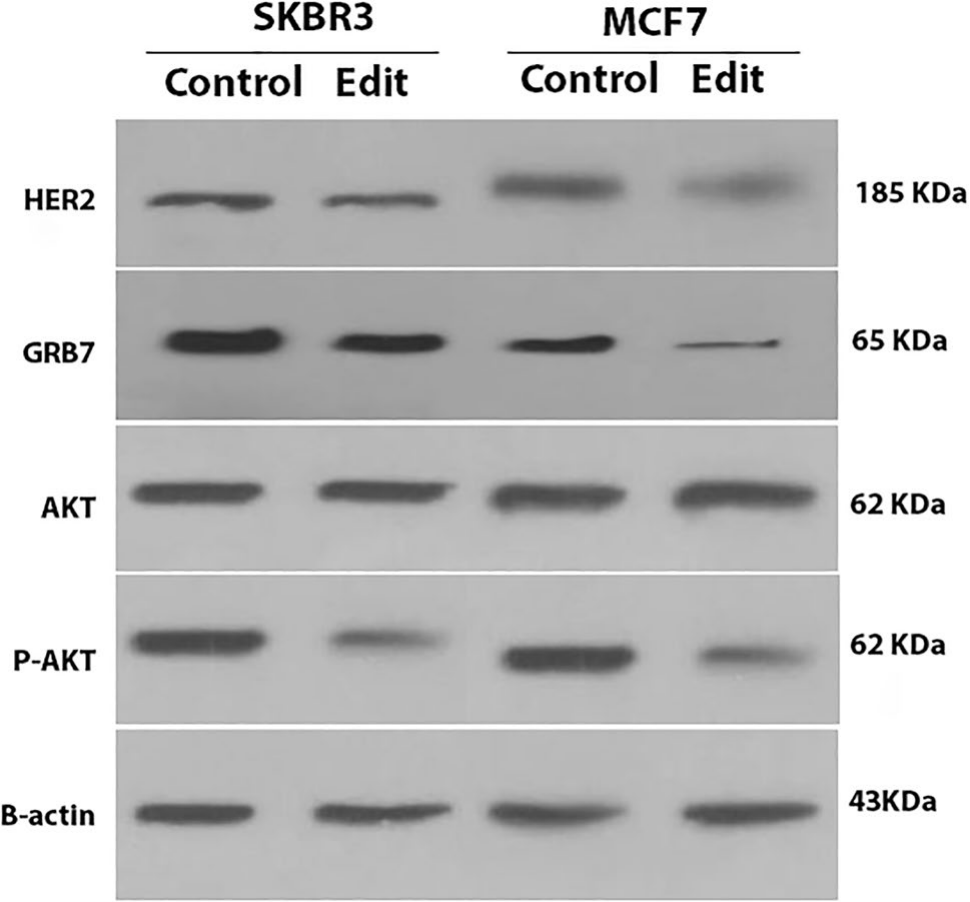
Western blot results. Genetic modification has substantially altered the proteins mentioned. HER2, GRB7, and p-AKT proteins showed a considerable decrease in both SKBR3 and MCF7 cell lines. The decrease in the expression of the investigated proteins is generally more pronounced in MCF7 compared to SKBR3, which is probably due to the low copy number of HER2. B-actin protein is considered an internal control. *Note* The merged gels produced the resulting image. In this experiment, a Mini Vertical Electrophoresis Cell system with two 10 × 10 cm gels was used. After electrotransfer and visualization using ECL, the original images from 12 × 12 cm films were scanned. The SuperSignal® Molecular Weight Protein Ladder with eight bands confirmed the completeness and integrity of the films. The ladder facilitated the identification of membrane edges, and the films exhibited a consistent ladder pattern.

Western blot results revealed decreased expressions in HER2, GRB7, and p-AKT proteins in both SKBR3 and MCF7 cell lines, in edited cells (Fig. S4 and Table S2). However, p-AKT expression decreased more dramatically than HER2 in SKBR3 cells, while the opposite result was observed in the MCF7 cell line with HER2 protein (Fig. 5).

### The regulatory role of Her2-En1 on cell cycle, apoptosis, and invasion

**Figure 6:**
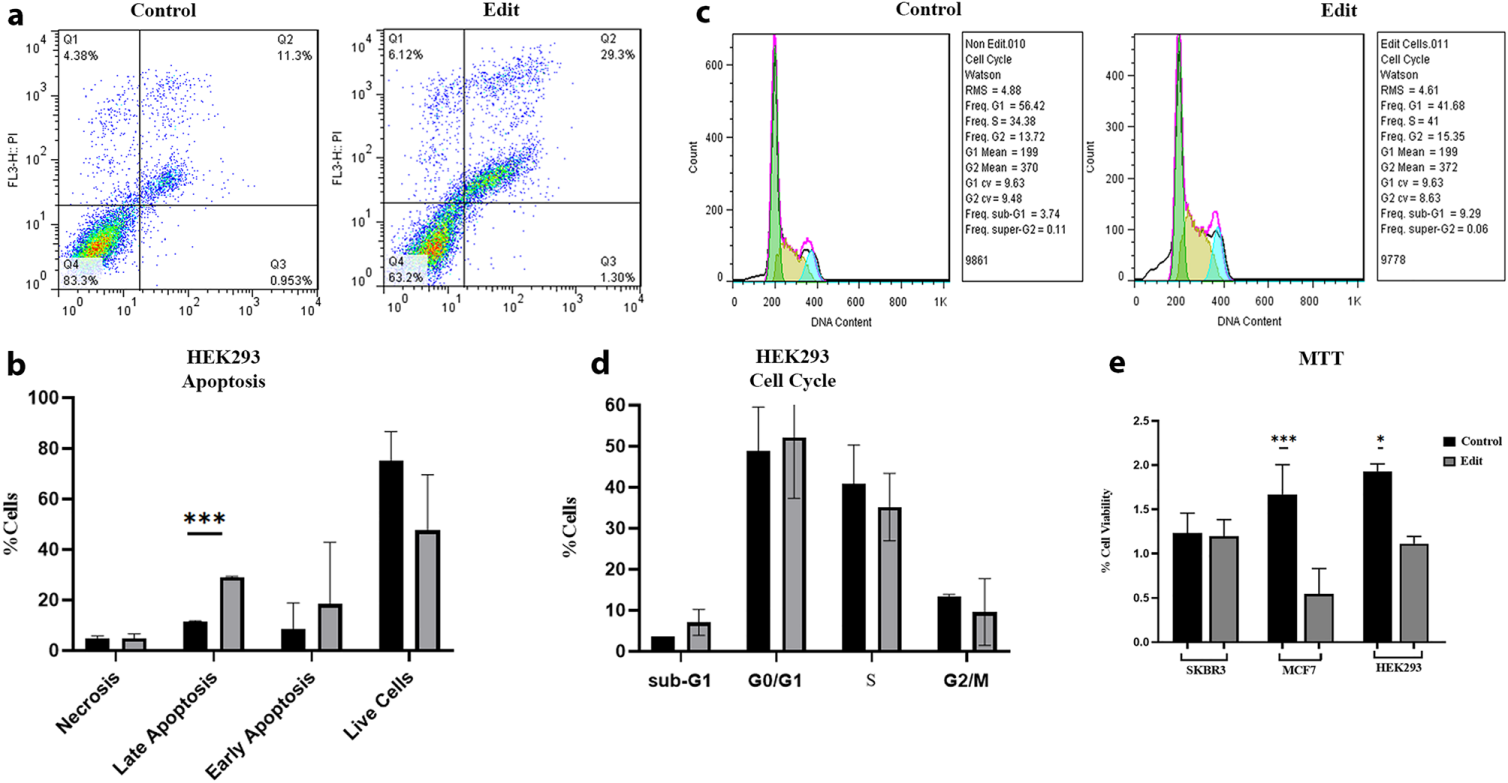
MTT and Flow cytometry results. (**a**–**d**) The results of apoptosis (**a**, **b**) and cell cycle (**c**, **d**) in HEK293. The results of apoptosis in the cell line HEK293 showed an increase in apoptosis and a decrease in live cells in edited cells. (**e**) MTT assay. The survival rate of the edited cells in two cell lines has decreased compared to the control cells. Probably, in SKBR3, due to the high copy number of HER2 and lack of complete editing of all copies, the percentage of cell survival has not been affected significantly. However, due to the presence of limited copies of the gene and its vital function, a higher percentage of MCF7 edited cells have suffered cell death compared to the control cells.

In general, our findings revealed a significant increase in the rate of apoptosis in edited cells, compared to the unedited ones (Fig. 6a,b), although, the results obtained from the SKBR3 cell line did not exhibit significant differences in terms of cell cycle and apoptosis (Fig. S5). In HEK293, the number of edited cells in the pre-G1 (sub-G1) and G1 phases of the cell cycle increased slightly, however, it insignificantly decreased in the G2 and S phases compared to the control cells (Fig. 6c,d). As shown in Fig. 6e, in the MTT assay, there was no significant difference between controlled and edited cells in the SKBR3 cell line, while significant differences in survival rates were observed in MCF7 (*P* Value: 0.0001) and HEK293 (*P* Value: 0.01) cell lines. In addition, Scratch test results in control and edited cells of SKBR3 and HEK293 showed no obvious differences between the two groups.

## Discussion

The importance of HER2 in the treatment of cancer lies in its role in tumor formation as well as its other roles as a marker for targeting various therapeutic agents^14^. Therefore, due to the importance of HER2 and its amplification and overexpression in cancers like breast cancer, the specific diagnosis and treatment of HER2+ cancers are necessary. Finding a marker related to HER2 amplification with implications in the diagnosis or treatment of HER2+ tumors is also of great clinical importance. Here, we predicted and then investigated the role of putative HER2 affecting Enhacer1 (so-called Her2-En1) as a regulatory element on the transcriptional balance of short and long variants of HER2 and other interacting genes in breast cancer.

At the heart of contemporary regulatory biology are DNase I-sensitive sites, many of which may act as cell-specific enhancers^11,15^. The discovery that many functional enhancers are transcription units and produce eRNAs had many implications for understanding gene regulation, growth, and disease^11^. eRNAs are transcriptional non-coding RNAs from the enhancers associated with super-enhancers and are also involved in the transcription of nearby genes. Specific conservation status compared to the flanking sequences, H3K27Ac mark, prediction of multiple *cis*-regulatory elements in the area, and finally detection of short transcripts belonging to the region through RT-PCR (Figs. 1, 2), all suggested the presence of putative regulatory region for the *HER2* expression. A similar approach has been reported for hunting the enhancer regions^11,15^.

CRISPR-Cas9 has been recently proposed as an efficient method for genetic engineering, enabling targeted deletion of genes with minimal off-target effects^16^. Previous studies using conditional knockout mouse models of HER2 in the mammary gland have shown that HER2 is required for normal morphogenesis of the mammary ducts^8^. In a study by Wang et al., the CRISPR system was successfully employed to target HER2 in HER2-amplified breast cancer cells resulting in the inhibition of cell proliferation^14^. To examine the functionality of the putative Her2-En1, we used CRISPR/Cas9 technology to partially or completely eliminate the regions, spanning, ~ 1000 or ~ 3000 bp of the human genomic DNA in three cell lines, respectively. Edit-test PCR verified the successful elimination of the chosen regions. Then, focusing on the cells (SKBR3, MCF7, & HEK293^17–20^) harboring ~ 1000 bp deletion in the putative Her2-En1 region, the RT-qPCR method indicated that regardless of the cell type, both short and long *HER2* transcript subtypes have been reduced, compared to the control groups. In a study by Liu et al., regions binding to an important transcription factor in HER2 enhancer named HGE were removed using the designed CRISPR-Cas9 system which led to HER2 downregulation^13^.

As well as the transcription level, the HER2 protein level was reduced following the elimination of the putative Her2-En1 region. Deletion of the Her2-En1 region was also followed by reduced expression level of GRB7 and PGAP3 genes in all tested cell lines, although it was not considerable for *PGAP3*. According to bioinformatic data obtained from UCSC and GeneCode, GRB7 and PGAP3 are genes that may interact with HER2 in the Her2-En1 region. PGAP3 (Post-GPI Attachment to Proteins Phospholipase 3) encodes a specific phospholipase for glycosyl phosphatidyl (GPI) that is initially located in the Golgi apparatus. The binding of proteins to the plasma membrane via GPI anchoring is involved in protein transferring and displacement^21,22^. The *PGAP3* expression level was affected by Her2-En1 elimination but not significantly. Another possible interacting protein GRB7 (Growth factor receptor protein 7), in our study, both its transcription and protein expression level have been affected by Her2-En1 elimination, is an adapter molecule that mediates the transmission of signals from the cell surface to various downstream signaling pathways^23^. GRB7 is overexpressed in breast cancer cell lines and primary breast tumors along with HER2 protein. GRB7 overexpression facilitates the phosphorylation process of HER2 and AKT in breast cancer cells overexpressing HER2, morphologically alters the cells, and increases xenograft tumor growth in nude mice. Thus, GRB7 overexpression plays an essential role in activating signal transduction and tumor growth in breast cancer cells by amplification of chromosome 17q11-21^23^. The oncogenic properties of HER2 are thought to be due to over-activation of downstream signaling cascades. In particular, signaling through the phosphoinositide 3-kinase (PI3K) -Akt pathway exerts multiple anti-apoptotic and growth-promoting effects^24^. The Akt or PI3K-Akt signaling pathway is a signal transduction pathway that promotes survival and growth in response to extracellular signals. Key proteins involved in this pathway are PI3K (phosphatidylinositol 3-kinase) and Akt (protein kinase B). Using phosphorylation of a variety of proteins, activated Akt mediates downstream regulatory effects, including cell survival, growth, proliferation, cell migration, and angiogenesis^25^. Akt also mediates G1-S phase progression and has an inhibitory effect on the phosphorylation and consequent degradation of cyclin D1 which in turn promotes the progression of the G1 phase in a positive feedback loop^26^. Fully activated PKB/Akt triggers diverse cellular functions involved in angiogenesis, metabolism, cell proliferation and survival, protein synthesis, transcriptional regulation, and apoptosis^27^. Cells overexpressing HER2 (e.g., adenocarcinoma cell line SKBR3) significantly activate long-term downstream pathways such as PI3K/AKT compared to cells with less expression of HER2 (such as MCF10A)^2^. Amplification and overexpression of HER2 protein are important factors in predicting clinical sensitivity to anti-HER2 therapies in patients with breast, gastric, and esophageal cancers^28^. In the current study, CRISPR-based targeting of Her2-En1 considerably reduced HER2 and its downstream p-AKT protein expression level. Our findings indicated that the Her2-En1 enhancer region likely is a positive regulator of the AKT activation and also showed the importance of this enhancer in the *HER2* and its effect on downstream signaling pathways, including the PI3K/Akt pathway. Nencioni A et al. identified a negative feedback loop mediated by Akt which is responsible for suppressing GRB7. They also observed that GRB7 was inhibited by the PI3K-Akt pathway. Thus, positive regulation of GRB7 as a result of inhibition of tyrosine kinase HER2 and EGFR will probably inactivate the PI3K-Akt signal transduction cascade. The rise of GRB7 seems to be a relatively premature event, which is well recognizable in the early hours after treatment or editing^24^. In this study, we observed a decrease in GRB7 protein in parallel with the reduction of HER2 (Fig. 5).

Her2-En1 deletion effect on the cell cycle progression marker gene expression was investigated through RT-qPCR. Results indicated that the BAX/BCL2 ratio which is an indicator of apoptosis level^29^, has been considerably elevated in both breast cancer cell types, and partly in HEK293 cells. Consistently, *C-myc* and *cyclin D1* oncogenes expression levels have been reduced in MCF7 and HEK293 cells, respectively. Also, *Rb* expression level has been reduced in SKBR3 and HEK293 cells. All of these RT-qPCR results indicated that the Her2-En1 region exerts its oncogenic effect on the cell cycle-related marker genes^30–32^ in these cell lines.

At the cellular level, Her2-En1 deletion partially affected cell cycle progression in all tested cell lines. This deletion resulted in a significantly increased number of late apoptotic HEK293 cells (P: 0.0003). It also resulted in an elevated number of Sub G1 cells and a reduced number of live and proliferative HEK cells consistent with the role of HER2 in cellular signaling^33^.

In summary, we showed that targeting Her2-En1 by CRISPR/Cas9 resulted in a reduction of HER2 expression at both RNA and protein levels. Although the knockout of the predicted enhancer affected the variants in cell line groups, differences were observed at various levels, such as the survival rate of cells post-editing. The difference in the HER2 gene's expression status between these cell lines may play a role. Different mechanisms or compensatory effects by multiple variants and the varying number of HER2 copies (Fig. S6 and Table S3) could potentially weaken the impact of editing on gene expression. However, there is no specific information about the effectiveness of bypassing the editing consequences in expression in HER2+ cell lines. It is only mentioned that increasing some transcript variants of HER2 in HER2-positive breast carcinomas causes resistance to anti-HER2 therapy^34^. Moreover, different HER2 signaling pathways may affect the editing. Once a ligand binds to the extracellular domain, the HER protein will dimerize and result in autophosphorylation of the intracellular domain, which in turn interacts with other signaling molecules that initiate the activation of a variety of downstream signaling pathways involved in cell proliferation, survival, and opposes apoptosis^35^.

RT-qPCR and Western blotting results also showed transcriptional changes in GRB7 as described earlier. Expression changes in genes involved in apoptosis, cell cycle, and proliferation are also presented in the results section (Figs. 4, 6). Dysregulation of the expression ratio of genes involved in apoptosis, cell cycle control, and cell proliferation (for example downstream of *HER2*) has been proven to be an important factor that can lead to uncontrolled cell proliferation and the development of many types of cancers including breast cancer^2^. Further investigation is required to determine the exact molecular events involved. Overall, the results of this study indicated the possible regulatory role of the Her2-En1 region as an enhancer in the HER2 gene.

## Conclusion

The genetic knockout of the desired promoter/enhancer region in HER2, resulted in a decrease in the expression of its variants in studied cell lines, indicating an important regulatory role of this region possibly as an HER2 enhancer/promoter. The Western blot analysis demonstrated a decrease in the translation of HER2, GRB7, and P-AKT proteins. Furthermore, alterations in the gene expression patterns were observed in the previously mentioned PGAP3 and GRB7 genes, which are believed to potentially interact with HER2. In this study, we demonstrated a substantial decrease in *GRB7*, which is located at the HER2 locus, however, no transcriptional change in *PGAP3* was observed. In addition, expression changes were also revealed in genes involved in cell cycle and cell proliferation after editing. Flow cytometry results also emphasized the importance of the Her2-En1 region in the cell cycle and apoptosis. Our findings suggest a *cis*/*trans*-regulatory role for this region, which is also expressed as an eRNA. generally, a deep understanding of the molecular implications of HER2 inhibition seems to be critical for designing therapeutic strategies to overcome drug resistance and improve clinical outcomes. Here, considering its effect on HER2 and GRB7 expression, Her2-En1 is suggested as another potential target for dealing with breast cancer therapy.

## Materials and methods

### Bioinformatics analysis

UCSC genome browser was used for browsing the genomic area around Enhacer1-Her2 located within the *HER2* sequence at 17q12 (Fig. 1).

### Design of oligonucleotides

The oligonucleotides used in PCR experiments were designed using oligo analyzer software and analyzed using NCBI primer-blast database. The primers were synthesized by Metabion (Metabion International AG, Germany). The sequences of the primers are provided in Table 1. The gRNAs used in this study were designed by CRISPOR software (http://crispor.org/).

**Table 1:** gRNAs and primers designed in this study.

Gene symbol	Sequences
Guide1: gRNA1 (HER2)	Sense: CACCGCACTTGTATCCTAACCATG Antisense: AAACCATGGTTAGGATACAAGTGC
Guide2: gRNA2 (HER2)	Sense: CACCGTCCCGGTGGGCGGTAGAGA Antisense: AAACTCTCTACCGCCCACCGGGAC
Guide3: gRNA3 (HER2)	Sense: CACCGCAGGAGAATCACTCGAATCC Antisense: AAACGGATTCGAGTGATTCTCCTGC
Guide4: gRNA4 (HER2)	Sense: CACCGCCCTGATCCTAACATCCCGA Antisense: AAACTCGGGATGTTAGGATCAGGGC
PX459	R: AGCCATTTGTCTGCAGAATTGG
Variant 1 HER2 Isoform a	F: GAGCCGCAGTGAGCACCAT R: AGCAGGTAGGTGAGTTCCAG
Variant 2 HER2 Isoform b	F: AGAGGCGATAGGGTTAAGGG R: GTGGTGAACAGGACAGCAAA
Primers for different HER2 Variants	F: TTCACCCACCAGAGTGATGTG R: ACATTCAGAGTCAATCATCCAACATT
HER2 primers (deletion test)	F1: CATTCCTGAGATGTGGGTAAGAG R1: TAACTGTAGGTCCCTCCCTCTAC F2: TAGCTGGATGTGGTGGTGCATG R2: GGTGGAGTTTTGTACAGGTATC
eRNA-HER2	F1: AAAGTGAGGTGAGTCGCAG R1: CCAAGCTTGCCAGACACTC F2: TCTCTACCGCCCACCGGGA R2: TAACTGTAGGTCCCTCCCTCTAC
U6 promoter	F: GAGGGCCTATTTCCCATGATT
Copy number primers	F: AAACTCTCTACCGCCCACCGGGAC R: TAACTGTAGGTCCCTCCCTCTAC
GAPDH	F: TCACTGTTCTCTCCCTCCG R: CATTGATGGCAACAATATCC
PGAP3	F: CGGTTGGTCCTGCTAGCT R: TCCAGCCTGCTAGACTCATG
GRB7	F: GTCCTCTCTTTGTGCCACCT R: CAGCAAGCACGGCAGGAT
P21	F: CCAGCATGACAGATTTCTACCAC R: GGCCAGGGTATGTACATGAGGAG
C-myc	F: CTCCTACGTTGCGGTCACAC R: CGGGTCGCAGATGAAACTCT
Rb	F: TCTGTGGATGGAGTATTGGGAGG R: TCACATGTCCTTTCCAATTTGCTG
Cyclin- D1	F: CCCTCGGTGTCCTACTCCAAA R: GAAGACCTCCTCCTCGCA
BCL2	F: GATACTGAGTAAATCCATGCAC R: AGTGTTGCAGAATATCAGCCAC
BAX	F: GGAGCTGCAGAGGATGATTGCC R: TCCCGCCACAAAATGGTCACG

### Cloning

The main steps of cloning including preparation of insert fragment, enzymatic digestion, ligation, transformation, investigation of transformed colonies by colony check PCR, culture of positive colonies, plasmid extraction, and confirmation of vector inserts were performed based on the routine protocol in Dr. Mowla’s lab. Briefly, gRNAs (two gRNAs targeting intron 3, exon 4, and intron 4 and two gRNAs targeting intron 4 of HER2) were cloned in PX459.v2 and rec-eCas9 vectors. TRE-KRAB-dCas9-IRES-GFP construct was also used for the desired sequence knock-down. The accuracy of the extracted constructs (GeneAll, South Korea) was further confirmed by direct sequencing (ABI 3500, USA).

### Cell culture

SKBR3, an adenocarcinoma-derived breast cancer cell line with high amplification and overexpression of *HER2* (HER2-positive), MCF7, an invasive ductal cell carcinoma derived from a breast cell line with low *HER2* expression (HER2-negative), and HEK293, a human embryonic renal cell line with moderate *HER2* expression were obtained from Iranian biological resource center (IBRC, Iran). The cells were cultured in DMED/F12 (Thermo Fisher Scientific, USA), supplied with 10% FBS (Thermo Fisher Scientific, USA), and 1% penicillin/streptomycin (Bio Basic, Canada) followed by incubation at 37 °C with 5% humidified CO2.

### Transfection of cells and predicted enhancer knockout using CRISPR/Cas9 system with Cas9 and gRNA vectors

To identify predicted enhancer regulatory effects, we designed four different gRNAs to target almost 1000–3000 bp for knocking out and knocking down this region, using the CRISPR/Cas9 system. Following the cell culture, about 100,000 cells were seeded in each well of a 12-well plate, and gRNA-containing vectors were transiently transfected into cells by Turbofect solution (Invitrogen, USA), 24 h later. Non-genetically modified parent cells were also used as control cells to compare the gene expression profiles. Then, the entire medium was changed the next day and the positive control plasmid (PEGFP-C1, Addgene, USA) was checked under the fluorescent microscope (Olympus ix53, Japan). Selection was carried out by treating the cells with 1.5 μg/ml Puromycin (Sigma-Aldrich, USA), 24 h after transfection. Pooled cells were investigated for deletion of the desired sequences by PCR (Edited-Test), using flanking primers. To this end, we extracted the cellular DNA using a GeneAll kit (South Korea) to ensure genetic modification. Following the PCR-confirmed editing, total RNA from control and edited cells was extracted using a TRIZOL reagent according to the manufacturer’s instructions (Thermo Fisher Scientific, USA).

### Gene expression assay by semi-quantitative RT-PCR and quantitative RT-qPCR

To confirm the inhibition of mRNA expression related to variants 1 and 2 of HER2 and other studied genes listed in Table 1, cDNA synthesis was performed on total RNAs extracted from edited and un-edited cells, using the manufacturer’s instructions (Thermo Fisher Scientific, USA). The synthesized cDNA was subsequently used as a template for PCR, where GAPDH was used as an internal control. PCR products were finally electrophoresed on 1.5% agarose gel. RT-qPCR technique was used to quantify the expression of the candidate genes, using primers listed in Table 1.

### Western blotting

Harvested cells were lysed with RIPA buffer (Sigma-Aldrich, USA) according to the manufacturer’s instructions. Protein concentrations in cell lysates were quantified using the Bradford assay. Western blotting was performed for HER2, GRB7, and AKT/PAKT proteins in SKBR3 and MCF7 cell lines. The methodology is summarized as follows: 30–50 μg of total protein for each sample was electrophorized on SDS-PAGE and blotted on PVDF membrane (Sigma-Aldrich, USA) afterward. After blocking with albumin, the membrane was incubated with anti-HER2, AKT/pAKT, and β-actin antibodies overnight at 4 °C. The membrane was then incubated with HRP-conjugated secondary antibody for 1 h at RT before visualizing with ECL kit (Lumigen, USA). The primary and secondary antibodies used in this study were as follows: Akt1 (B-1): sc-5298, β-Actin (C4): sc-47778, p-Akt1/2/3 (B-5): sc-271966, Neu (F-11): sc-7301, GRB7 (A-12): sc-376069 [1:300 mouse monoclonal antibody (Santa Cruz Biotechnology, Inc.)], m-IgGκ BP-HRP: sc-516102, mouse anti-rabbit IgG-HRP: sc-2357 (Santa Cruz Biotechnology, Inc.).

### MTT assay

MTT assay was used to determine the percentage of survival and cell death after transfection of the studied cells. First, 200 µl of medium containing about 10,000 cells were seeded in a 96-well plate. A cell-free well was also used as the control. To measure cell mortality, 40 μl (10% of medium volume on cells) of MTT solution (Sigma, USA) with a concentration of 5 mg/ml made in PBS was added to each well, 24 h later. The foil-wrapped plate was returned to a 37 °C incubator for 4 h. The medium containing MTT was then discarded and 200 μl of DMSO, as formazone solvent, was added to each well. After homogenization by incubating for 20 min in a shaker incubator, the light absorption at 570 nm was read by an ELISA reader (Biotek ELx800, USA). The higher light absorption rates proportionally indicate higher cell survival rates. A standard graph of survival cells in each cell line was drawn using the obtained absorbents.

### Flow cytometry

Distribution of the treated and untreated MCF7, HEK293, and SKBR3 cell lines within different phases of the cell cycle, as well as the percentage of the apoptotic and necrotic cells, were calculated by the BD FACSCalibur Flow cytometer (BD Biosciences, San Jose, CA, USA).

### Statistical and data analysis

Statistical analyses were carried out using Student T-test and ANOVA, to investigate the significance of the observed differences in measured variables, between edited and non-edited cells. All tests were conducted in at least 2 biological replicates and values were reported as mean ± standard deviation. *P* values below 0.05 were considered statistically significant. All graphs were drawn with GraphPad Prism 9.

### Ethical statement

This research did not involve human participants or using human biological samples. It also did not include experimenting on live animals. The in vitro experiments on commercial cell lines were approved as a Ph.D. thesis proposal, by Tarbiat Modares Research Ethics Committee (reference number: IR.MODARES.REC.1399.067). This manuscript has been read and approved by all authors. This paper is unique and is not under consideration by any other publication and has not been published elsewhere. The authors and peer reviewers of this paper report no conflicts of interest. The authors confirm that they have permission to reproduce any copyrighted material.

### Supplementary Information

Supplementary Information.

## Data Availability

The datasets used and analyzed during this study are available by the Corresponding author on reasonable request.
