# Supplementary material for: Functional analysis of a putative HER2-associated expressed enhancer, Her2-Enhancer1, in breast cancer cells
Source: Sci Rep. 2023 Nov 9;13:19516. doi: 10.1038/s41598-023-46460-x (PMC10636096; doi:10.1038/s41598-023-46460-x)
Supplement: Supplementary file 1 — Supplementary Information. [file 41598_2023_46460_MOESM1_ESM.pdf]

Legends

Supplementary Figure 1. Clustered Interactions of GeneHancer Regulatory Elements and Genes. a. Gene targets for GeneHancer: GH17J039694 in GeneCards. b. Interactions between GeneHancer regulatory elements and genes in UCSC.

Supplementary Figure 2. Confirmation of gRNAs cloned in vectors and deleted area through sequencing. **a.** Sequencing result of gRNAs designed for knockout. **b.** Sequencing result of the deleted area with 995 bases removed from the original enhancer sequence (Her2-En1). **c.** Sequencing of the deleted region with edit-test primers displayed at UCSC.

Supplementary Figure 3. Quantification of Gel Bands. **a, b**. The gel and table display the values of variants in two modes, controlled and edited. These values were calculated using ImageJ software. **c, d**. Both controlled and edited HER2 variants are shown in an RT-PCR gel. The corresponding table of calculated values is also presented. (Note: the second repetition is not placed in the original manuscript.) **e.** Column chart of the gel bands.

Supplementary Figure 4. Western Blot Quantification. **a.** Western Blot Gel. **b.** Table of Values and Quantification in Two Groups: Control and Edited, in SKBR3 and MCF7 Cell Lines. **c.** Column chart of the gel bands.

Supplementary Figure 5. The results of apoptosis and cell cycle in SKBR3 and MCF7. **a & b.** Illustrates the cell cycle and apoptosis in SKBR3 cells, with a representing the control group and b representing the edited group. In general, our results indicate a higher occurrence of apoptosis in the edited SKBR3 cells compared to the control cells. **c & d.** Depicts the cell cycle in MCF7 cells, where c represents the control group and d represents the edited group. Notably, there were no significant differences observed between the two groups in terms of cell cycle progression. **e.** Section e Presents a bar graph portraying the cell cycle distribution in MCF7 cells. The results indicate that although there were no apparent variations between the two groups, the number of edited cells in the pre-G1 phase initially increased and then decreased during the S phase.

Supplementary Figure 6. Post-editing measurement of HER2 (GH17J039694) DNA copy number in cells with the help of Standard Curve. **a.** Standard Curve Diagram: The diagram illustrates the standard curve used for measuring the HER2 DNA copy number in cells. **b.** The column chart of the copy numbers: This chart displays the calculated number of copies using the formula X (=copy number) = 38.45 **-** Y (=CT mean of samples)/3.731. **c.** Melting curve. **d.** Sample Copy Number: This graph represents the measured copy number of HER2 DNA in the samples. **e.** Plasmid Copy Number: The Table showcases the copy number of

the HER2 DNA plasmid used as a reference. **f**. PCR Efficiency: This graph demonstrates the efficiency of the Quantitative Polymerase Chain Reaction (qPCR) method used in the experiment.

Note: The copy number of desired DNA fragments of GH17J039694 as a regulatory element in *HER2* were evaluated in SKBR3, MCF7, and HEK293 cells using quantitative real-time PCR. Serial dilutions of plasmids harboring HER2 carried out absolute quantification. In this study, the number of gene copies per 100 ng of DNA of cells (control and edited), was calculated from 10 to 10^7 copies. The standard curve revealed a linear relationship between CT and the initial amount of DNA, so the concentrations were derived from standard curves based on CT values. Seven series of 10-fold dilutions of HER2 plasmid were prepared for plotting the standard curve.

Supplementary Figure 7. Original Unprocessed Images. **a.** HER2- eRNA Expression (HER2- Expressed Enhancer) in Three Cell Lines: The image shows the expression of HER2- eRNA using primers for a specific region of Enhancer DNA in three different lines. **b.** PCR Edit-Test: Both unedited and edited bands are displayed in this image. The unedited band (1239 bp) and the edited band (241 bp) represent a partial knockout scenario. **c.** Complete Knockout: This image demonstrates a complete knockout, where the edited band appears at 482 bp. Additionally, an unedited band of approximately 1000 bp can also be observed. NTC refers to the non-template control. **d.** RT-PCR with GAPDH as an Internal Control: HER2 variants 1 (214 bp) and 2 (156 bp) were analyzed in SKBR3 and MCF7 cell lines. The edited variants in the cell lines are indicated by wells 6, 8, 10, and 12 from the left. **e.** Original western blotting images. Original Western blotting images depicting the expression levels of the proteins mentioned in the manuscript. The merged gels produced the resulting image. **Note:** The images in parts b and c were taken from two distinct gels, which were run at different time points and then the photos were merged. Before sample loading, the gels were cut. Gel images were processed very limitedly in PowerPoint in the original manuscript. This Supplementary Figure encompasses the unprocessed original images of Figures 2b and 3b-d as presented in the initial manuscript.

Supplementary Figure 8. Unprocessed images of Western radiography films. In this experiment, we utilized the Mini Vertical Electrophoresis Cell system, comprising two gels measuring 10 x 10cm each. Subsequently, during the electrotransfer phase and the visualization step using ECL, a PVDF membrane measuring 12 x 12cm and radiography film were employed, respectively. Consequently, all protein bands were successfully transferred onto the membrane and subsequently onto the films. The original scanned images were acquired from the final films with dimensions of 12 x 12cm. Furthermore, the completeness and integrity of the films were confirmed by employing the Chemiluminescence ladder, specifically the SuperSignal® Molecular Weight Protein Ladder with catalog number 84785. This ladder consists of eight bands with varying molecular weights ranging from 20-150 KDa. It is worth noting that all eight bands are

visible in the original images, which further confirms the unaltered nature of the films. Additionally, due to the consecutive probes and stripping processes involved, the films exhibit a consistent ladder pattern. Notably, some of these films, indicated by the arrows, display four distinct lanes, which, along with the presence of the four-lane and eight-band ladder, facilitate the identification of the membrane edges.

Supplementary Table 1. Quantification of the gel bands. **a & b**. The tables, labeled Table a and Table b, present the values of variants in two different modes: controlled and edited. These values were calculated using the ImageJ software.

Supplementary Table 2. The quantification of Western Blot results. This Table showcases the values obtained for the control and edited groups in SKBR3 and MCF7 cell lines.

Supplementary Table 3. Post-editing measurement of HER2 (GH17J039694) DNA copy number in cells, assisted by a Standard Curve. **a.** Sample copy number. **b.** Plasmid copy number. **c**. PCR efficiency.

a

	GeneHancer (GH) Identifier	GH Type	GH Score	GH Sources	Gene Association Score	Total Score	TSS distance (kb)	Number of Genes Away	Size (kb)	Transcription Factor Binding Sites	Gene Targets
⊖	GH17J039694	Promoter/Enhancer	1.7★	RefSeq, Ensembl, ENCODE, CraniofacialAtlas, dbSUPER	0.45	0.78	+7.9	2	2.9	122 TFs ⊖ KLF6 DNMT3B ...	20 genes ⊖ PGAP3 ...

Gene targets for GeneHancer: PGAP3 LN812234 CDK12 CWC25 LOC105371762 SNORA21 ENSG00000266469 MED1 MLLT6 SMARCE1 RPL23 MIEN1 IGFBP4 GRB7 NEUROD2 RPL19 ORMDL3 LRRC3C lnc-PNMT-1 ERBB2

Genomic Location: chr17:39694339-39697219 (GRCh38/hg38) chr17:37850592-37853472 (GRCh37/hg19)

b

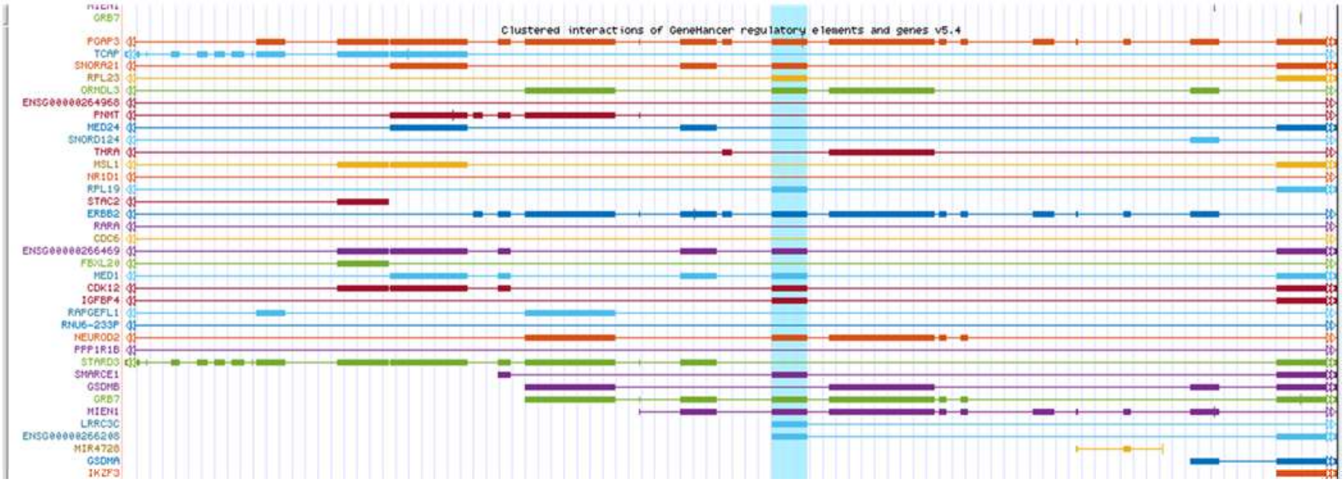

Fig S1.

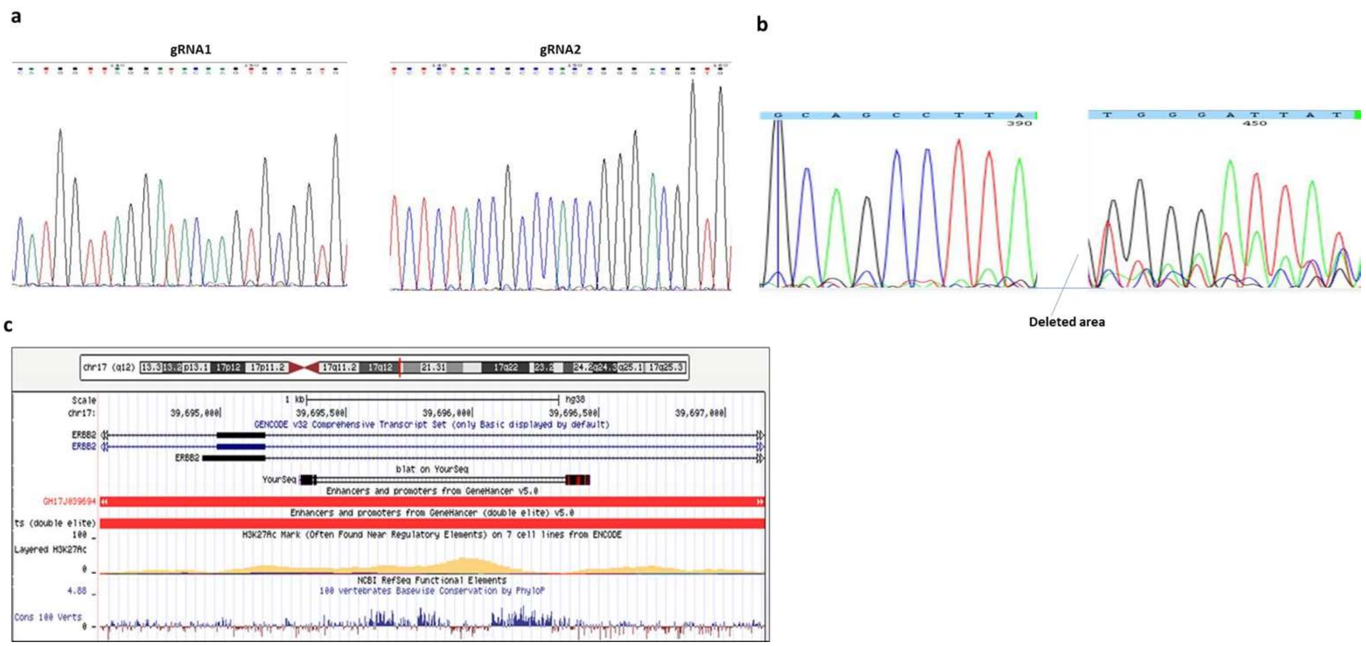
a
gRNA1
gRNA2
b
390
450
Deleted area
c
chr17 (q12)
Scale
1 kb
hg38
chr17:
39,695,000
39,695,500
39,696,000
39,696,500
39,697,000
GENCODE v32 Comprehensive Transcript Set (only Basic displayed by default)
ERBB2
ERBB2
ERBB2
blat on YourSeq
YourSeq
Enhancers and promoters from GeneHancer v5.0
GH17J039694
Enhancers and promoters from GeneHancer (double elite) v5.0
ts (double elite)
100
Layered H3K27Ac
H3K27Ac Mark (Often Found Near Regulatory Elements) on 7 cell lines from ENCODE
0
NCBI RefSeq Functional Elements
4.88
100 vertebrates Basewise Conservation by PhyloP
Cons 100 Verts
0

Fig S2.

a

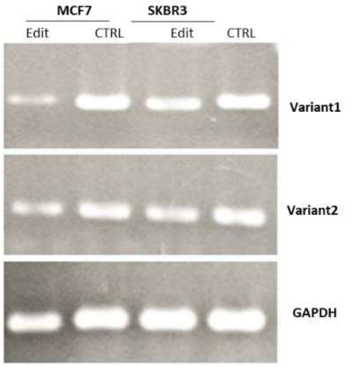

b

Sample type	Variable	Group	Target density	Target density (fold change)
SKBR3	GAPDH	Edit	0.049	
		CTRL	0.049	
	Variant1	Edit	0.031	0.794871795
		CTRL	0.039	
	Variant2	Edit	0.033	0.846153846
		CTRL	0.039	
MCF7	GAPDH	Edit	0.041	
		CTRL	0.048	
	Variant1	Edit	0.021	0.702439024
		CTRL	0.035	
	Variant2	Edit	0.027	0.87804878
		CTRL	0.036	

c

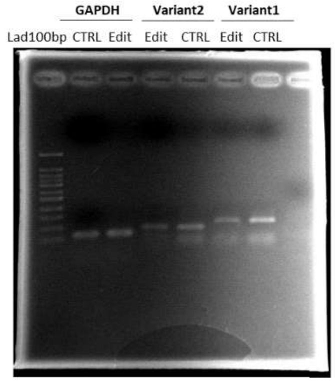

d

Sample type	Variable	Group	Target density	Target density (fold change)
SKBR3	GAPDH	Edit	53677.638	
		CTRL	50792.952	
	Variant1	Edit	51602.902	0.817342
		CTRL	59742.065	
	Variant2	Edit	38339.304	0.773386
		CTRL	46909.198	

e

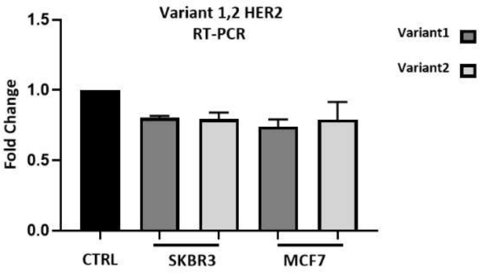

Fig S3.

a

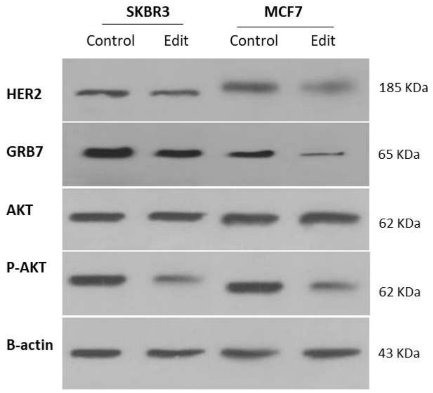

b

Sample type	Variable	Group	Target density	Target density (fold of control)
Human cell line (SKBR3)	HER-2	CTRL	9912.539	1
		Edit	7089.054	0.715160263
	P-AKT	CTRL	12346.296	1
		Edit	5568.246	0.451005387
	AKT	CTRL	12310.953	1
		Edit	11632.782	0.9449132
	GRB7	CTRL	22787.602	1
		Edit	15448.388	0.677929516
	B-actin	CTRL	10323.296	1
		Edit	10362.953	1.003841506

Sample type	Variable	Group	Target density	Target density (fold of control)
Human cell line (MCF7)	HER-2	CTRL	10182.974	1
		Edit	6555.024	0.643723926
	P-AKT	CTRL	10546.539	1
		Edit	6612.296	0.626963594
	AKT	CTRL	11321.368	1
		Edit	13023.903	1.150382445
	GRB7	CTRL	12162.489	1
		Edit	3115.255	0.256136306
	B-actin	CTRL	8185.832	1
		Edit	8414.347	1.027915916

c

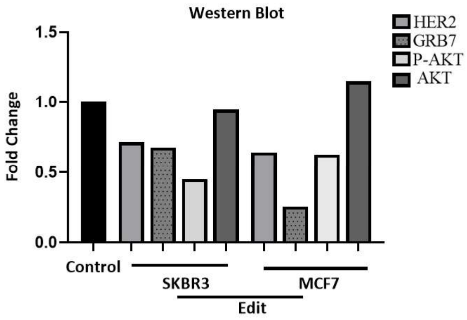

Fig S4.

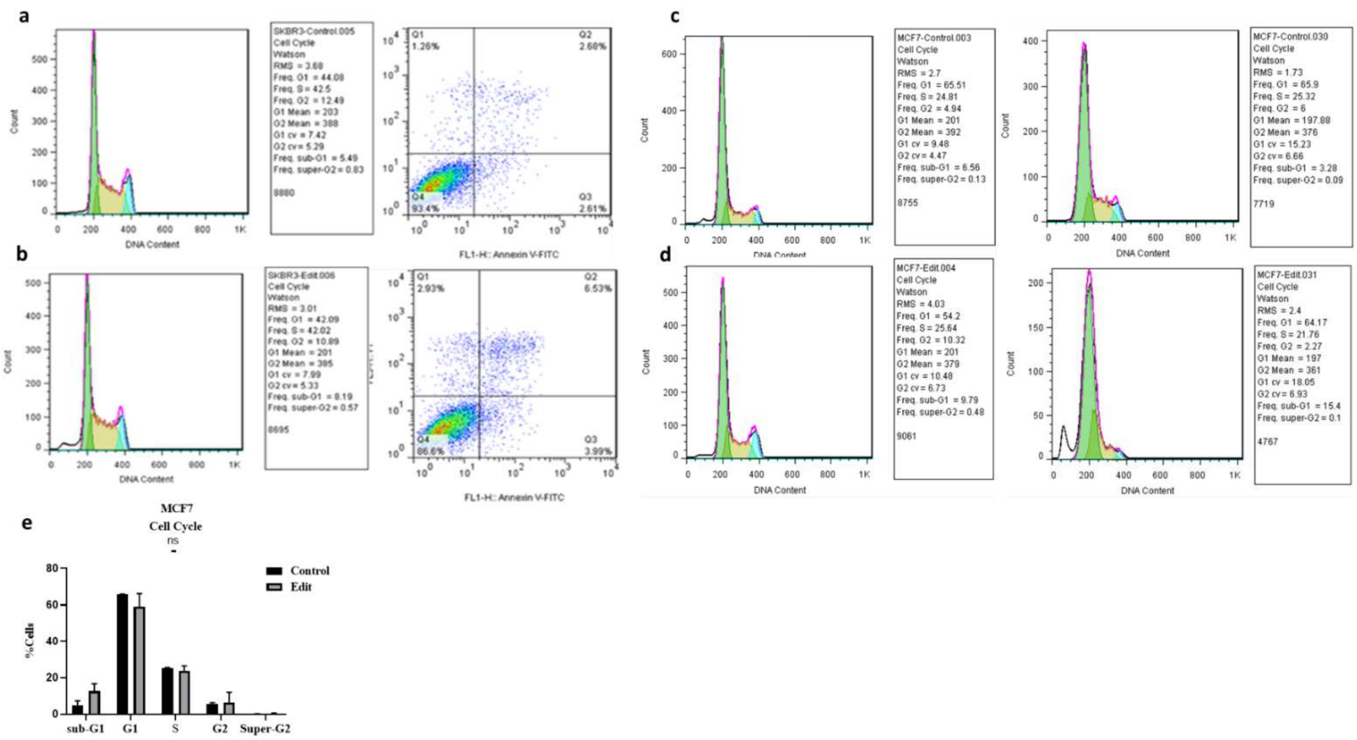
a
b
c
d
e
SKBR3-Control.005
Cell Cycle
Watson
RMS = 3.68
Freq. G1 = 44.08
Freq. S = 42.5
Freq. G2 = 12.49
G1 Mean = 203
G2 Mean = 388
G1 cv = 7.42
G2 cv = 5.29
Freq. sub-G1 = 5.49
Freq. super-G2 = 0.83
8880
SKBR3-Edit.006
Cell Cycle
Watson
RMS = 3.01
Freq. G1 = 42.09
Freq. S = 42.02
Freq. G2 = 10.89
G1 Mean = 201
G2 Mean = 385
G1 cv = 7.99
G2 cv = 5.33
Freq. sub-G1 = 8.19
Freq. super-G2 = 0.57
8695
Q1 1.26%
Q2 2.68%
Q4 93.4%
Q3 2.61%
Q1 2.93%
Q2 6.53%
Q4 86.6%
Q3 3.99%
FL1-H:: Annexin V-FITC
MCF7-Control.003
Cell Cycle
Watson
RMS = 2.7
Freq. G1 = 65.51
Freq. S = 24.81
Freq. G2 = 4.94
G1 Mean = 201
G2 Mean = 392
G1 cv = 9.48
G2 cv = 4.47
Freq. sub-G1 = 6.56
Freq. super-G2 = 0.13
8755
MCF7-Control.030
Cell Cycle
Watson
RMS = 1.73
Freq. G1 = 65.9
Freq. S = 25.32
Freq. G2 = 6
G1 Mean = 197.88
G2 Mean = 376
G1 cv = 15.23
G2 cv = 6.66
Freq. sub-G1 = 3.28
Freq. super-G2 = 0.09
7719
MCF7-Edit.004
Cell Cycle
Watson
RMS = 4.03
Freq. G1 = 54.2
Freq. S = 25.64
Freq. G2 = 10.32
G1 Mean = 201
G2 Mean = 379
G1 cv = 10.48
G2 cv = 6.73
Freq. sub-G1 = 9.79
Freq. super-G2 = 0.48
9061
MCF7-Edit.031
Cell Cycle
Watson
RMS = 2.4
Freq. G1 = 64.17
Freq. S = 21.76
Freq. G2 = 2.27
G1 Mean = 197
G2 Mean = 361
G1 cv = 18.05
G2 cv = 6.93
Freq. sub-G1 = 15.4
Freq. super-G2 = 0.1
4767
Count
DNA Content
MCF7
Cell Cycle
ns
Control
Edit
%Cells
sub-G1
G1
S
G2
Super-G2

Fig S5.

a

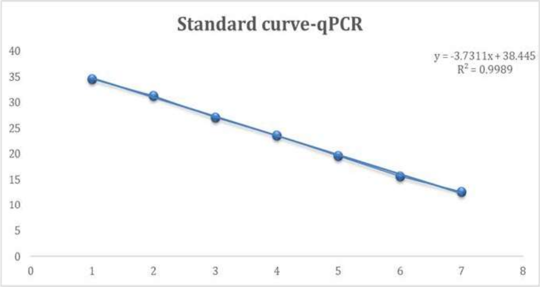

b

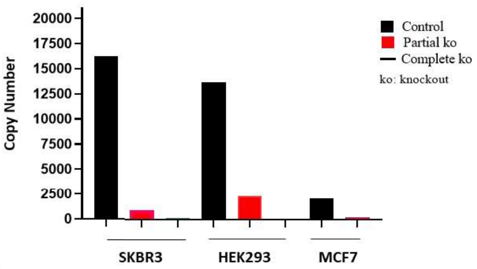

c

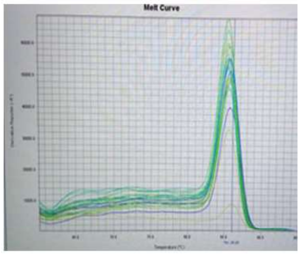

d

Sample Name (cell lines)	Mean CT	Log copy number in pool	Copy in pool
SKBR3 (control)	22.26	4.337863	21770.22054
SKBR3-EDIT1	27.3676	2.968937	930.9723331
SKBR3-EDIT2 (complete ko)	30.58	2.107957	128.2204919
MCF7 (control)	25.65	3.429284	2687.098517
MCF7-EDIT	29.695	2.345153	221.3874021
HEK293 (control)	23.7943	3.926644	8445.854923
HEK293-EDIT1	25.826	3.382113	2410.52985
HEK293-EDIT2 (complete ko)	37.07	0.368524	2.336275355

e

Standard copy number	Log standard copy number	Mean CT	Standard log copy in formula
10000000	7	12.615	6.922891373
1000000	6	15.72	6.090697113
100000	5	19.695	5.025327651
10000	4	23.635	3.969338801
1000	3	27.07	3.048698775
100	2	31.35	1.901583983
10	1	34.56	1.041247889

f

**y=mx+b**	**m= Slope**
	**b= intercept**
y = -3.7311x + 38.445	m=-3.7311
	b= 38.445
**PCR efficiency (E)**	**E=10^(-1/slope)**
E	1.853606206
**E%=(E-1)*100**	85.36062056

Fig S6.

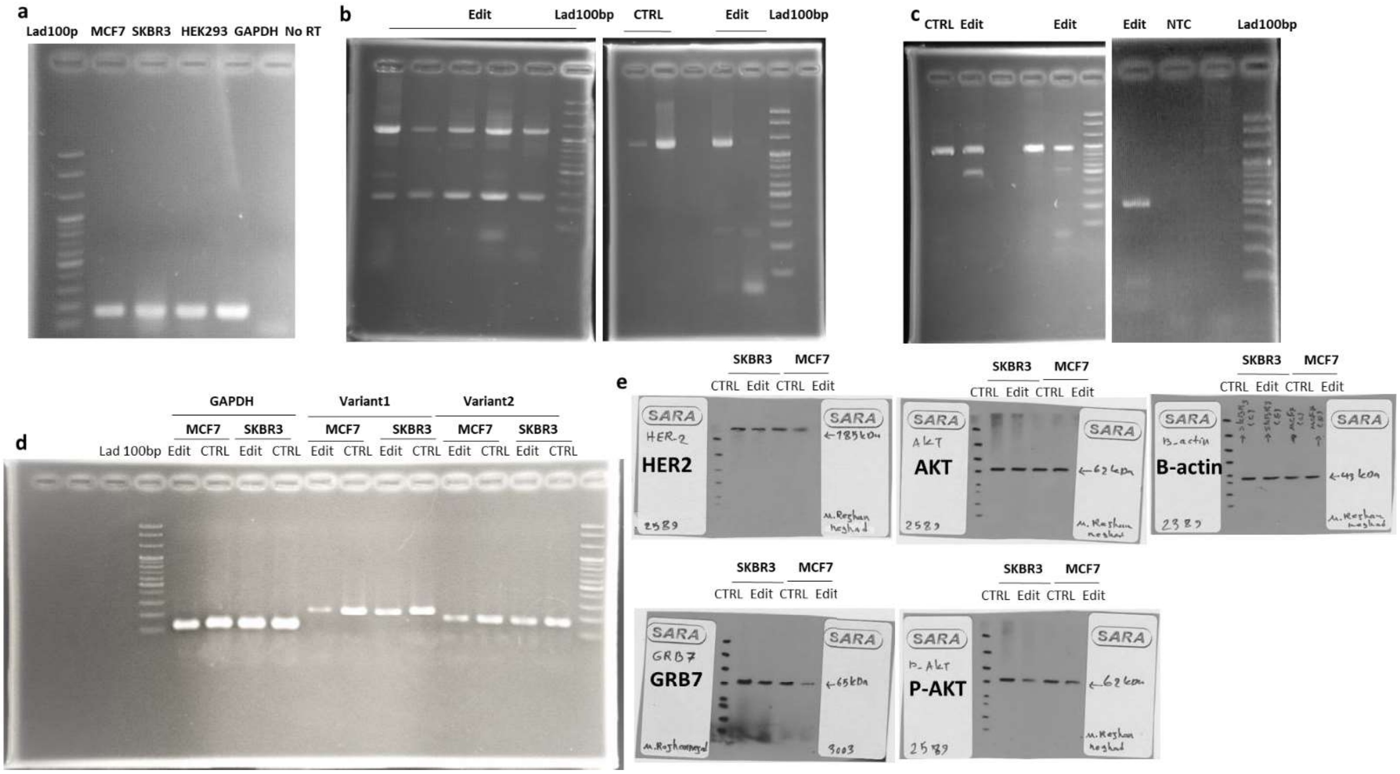
a
Lad100p MCF7 SKBR3 HEK293 GAPDH No RT
b
Edit
Lad100bp
CTRL
Edit
Lad100bp
c
CTRL Edit
Edit
Edit NTC
Lad100bp
d
GAPDH
Variant1
Variant2
MCF7 SKBR3 MCF7 SKBR3 MCF7 SKBR3
Lad 100bp Edit CTRL Edit CTRL Edit CTRL Edit CTRL Edit CTRL Edit CTRL
e
SKBR3 MCF7
CTRL Edit CTRL Edit
HER2
AKT
B-actin
GRB7
P-AKT

Fig S7.

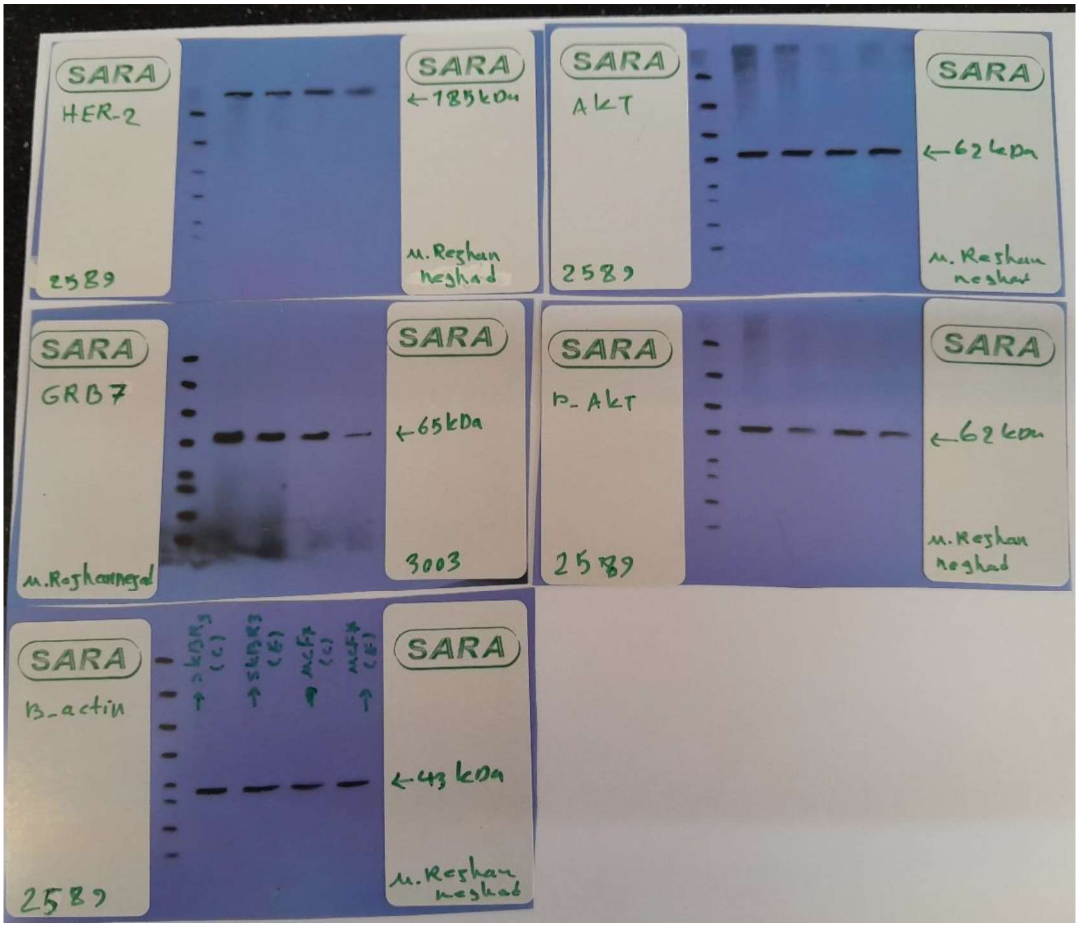
SARA
HER-2
←185kDa
2589
AKT
←62kDa
GRB7
←65kDa
3003
p-AKT
←62kDa
B-actin
←43kDa

Fig S8.

a

Sample type	Variable	Group	Target density	Target density (fold change)
SKBR3	GAPDH	Edit	0.049	
		CTRL	0.049	
	Variant1	Edit	0.031	0.794871795
		CTRL	0.039	
	Variant2	Edit	0.033	0.846153846
		CTRL	0.039	
MCF7	GAPDH	Edit	0.041	
		CTRL	0.048	
	Variant1	Edit	0.021	0.702439024
		CTRL	0.035	
	Variant2	Edit	0.027	0.87804878
		CTRL	0.036	

b

Sample type	Variable	Group	Target density	Target density
SKBR3	GAPDH	Edit	53677.638	
		CTRL	50792.952	
	Variant1	Edit	51602.902	0.817342
		CTRL	59742.065	
	Variant2	Edit	38339.304	0.773386
		CTRL	46909.198	

Table S1.

Sample type	Variable	Group	Target density	Target density (fold of control)
Human cell line (SKBR3)	HER-2	CTRL	9912.539	1
		Edit	7089.054	0.715160263
	P-AKT	CTRL	12346.296	1
		Edit	5568.246	0.451005387
	AKT	CTRL	12310.953	1
		Edit	11632.782	0.9449132
	GRB7	CTRL	22787.602	1
		Edit	15448.388	0.677929516
	B-actin	CTRL	10323.296	1
		Edit	10362.953	1.003841506
Sample type	Variable	Group	Target density	Target density (fold of control)
Human cell line (MCF7)	HER-2	CTRL	10182.974	1
		Edit	6555.024	0.643723926
	P-AKT	CTRL	10546.539	1
		Edit	6612.296	0.626963594
	AKT	CTRL	11321.368	1
		Edit	13023.903	1.150382445
	GRB7	CTRL	12162.489	1
		Edit	3115.255	0.256136306
	B-actin	CTRL	8185.832	1
		Edit	8414.347	1.027915916

Table S2.

a

Sample Name (cell lines)	Mean CT	Log copy number in the pool	Copy in pool
**SKBR3 (control)**	**22.26**	**4.337863**	**21770.22054**
**SKBR3-EDIT1**	**27.3676**	**2.968937**	**930.9723331**
**SKBR3-EDIT2 (complete ko)**	**30.58**	**2.107957**	**128.2204919**
**MCF7 (control)**	**25.65**	**3.429284**	**2687.098517**
**MCF7-EDIT**	**29.695**	**2.345153**	**221.3874021**
**HEK293 (control)**	**23.7943**	**3.926644**	**8445.854923**
**HEK293-EDIT1**	**25.826**	**3.382113**	**2410.52985**
**HEK293-EDIT2 (complete ko)**	**37.07**	**0.368524**	**2.336275355**

b

Standard copy number	Log standard copy number	Mean CT	Standard log copy in the formula
**10000000**	**7**	**12.615**	**6.922891373**
**1000000**	**6**	**15.72**	**6.090697113**
**100000**	**5**	**19.695**	**5.025327651**
**10000**	**4**	**23.635**	**3.969338801**
**1000**	**3**	**27.07**	**3.048698775**
**100**	**2**	**31.35**	**1.901583983**
**10**	**1**	**34.56**	**1.041247889**

c

**y=mx+b**	**m= Slope**
	**b= intercept**
**y = -3.7311x + 38.445**	**m=-3.7311**
	**b= 38.445**
**PCR efficiency (E)**	**E=10^(-1/slope)**
**E**	**1.853606206**
**E%=(E-1) *100**	**85.36062056**

Table S3.
